# Individual Immune-Modulatory Capabilities of MSC-Derived Extracellular Vesicle (EV) Preparations and Recipient-Dependent Responsiveness

**DOI:** 10.3390/ijms20071642

**Published:** 2019-04-02

**Authors:** Lambros Kordelas, Esther Schwich, Robin Dittrich, Peter A. Horn, Dietrich W. Beelen, Verena Börger, Bernd Giebel, Vera Rebmann

**Affiliations:** 1 Department of Bone Marrow Transplantation, University Hospital Essen, 45147 Essen, Germany; Lambros.Kordelas@uk-essen.de (L.K.); Dietrich.Beelen@uk-essen.de (D.W.B.); 2 Institute for Transfusion Medicine, University Hospital Essen, 45147 Essen, Germany; Esther.Schwich@uk-essen.de (E.S.); Robin.Dittrich@uk-essen.de (R.D.); Peter.Horn@uk-essen.de (P.A.H.); Verena.Boerger@uk-essen.de (V.B.); Bernd.Giebel@uk-essen.de (B.G.)

**Keywords:** mesenchymal stem/stromal cells (MSC), extracellular vesicles (EV), Graft-versus-Host-Disease (GvHD)

## Abstract

Treatment with extracellular vesicles (EVs) derived from mesenchymal stem/stromal cells (MSCs) have been suggested as novel therapeutic option in acute inflammation-associated disorders due to their immune-modulatory capacities. As we have previously observed differences in the cytokine profile of independent MSC-EV preparations, functional differences of MSC-EV preparations have to be considered. To evaluate the immune-modulatory capabilities of specific MSC-EV preparations, reliable assays are required to characterize the functionality of MSC-EV preparations prior to administration to a patient. To this end, we established an in vitro assay evaluating the immune-modulatory capacities of MSC-EV preparations. Here, we compared the efficacy of four independent MSC-EV preparations to modulate the induction of T cell differentiation and cytokine production after phorbol 12-myristate 13-acetate (PMA)/Ionomycin stimulation of peripheral blood mononuclear cells (PBMC) derived from six healthy donors. Flow cytometric analyses revealed that the four MSC-EV preparations differentially modulate the expression of surface markers, such as CD45RA, on CD4+ and CD8+ T cells, resulting in shifts in the frequencies of effector and effector memory T cells. Moreover, cytokine profile in T cell subsets was affected in a MSC-EV-specific manner exclusively in CD8+ naïve T cells. Strikingly, hierarchical clustering revealed that the T cell response towards the MSC-EV preparations largely varied among the different PBMC donors. Thus, besides defining functional activity of MSC-EV preparations, it will be crucial to test whether patients intended for treatment with MSC-EV preparations are in principal competent to respond to the envisioned MSC-EV therapy.

## 1. Introduction

In a variety of disorders, inflammatory processes are the pathological core, which lead to detrimental effects. One of these inflammatory processes is the “Graft-versus-Host-Disease” (GvHD) after allogeneic hematopoietic stem cell transplantation (alloSCT). Here, GvHD is the result of immune reactions of donor T cells against the patient’s tissue recognized as foreign [1]. Thus, therapeutic strategies of GvHD treatment aim at the inhibition or deletion of alloreactive donor T cells by conventional treatments, such as corticosteroids. In steroid-refractory cases of GvHD the application of mesenchymal stem/stromal cells (MSC) as immune cell therapy options have been evaluated as second-line treatment, however with varying results [2].

Recent data suggest that the beneficial immune-modulatory effect of MSCs derive rather from secreted vesicles than from MSC themselves [3,4]. Extracellular vesicles (EVs) are a heterogeneous population amongst others comprising exosomes, microvesicles, and apoptotic bodies [5]. EVs contain proteins, microRNA, mRNA, and lipids and play an important role in intercellular communication [6,7]. Depending on their cellular origin and cytokine cargo with either pro- or anti-inflammatory cytokines, EVs can exert immune-stimulatory or immune-suppressive functions, respectively [8]. MSC-EVs offer various advantages compared to the application of integral MSCs, as they are non-self-replicating and sterile application of EVs is easily feasible due to their small size by passing them through a 0.22 µm filter membrane. Our center applied for the first time MSC-EVs successfully in a patient with therapy-refractory cutaneous and intestinal acute GvHD IV° [9]. Indeed, MSC-EV administration showed significant effects in vitro and in vivo. In vitro, MSC-EVs reduced the production of pro-inflammatory cytokines of patient-derived peripheral blood mononucleated cells (PBMCs) and NK cells. The in vivo capability of the patient’s PBMCs to release proinflammatory cytokines was impaired and a decline in proinflammatory cytokines was observed in the blood of the patient during the course of the MSC-EV application. Consistent with these findings, the cutaneous and intestinal GvHD symptoms of the patient improved significantly. As several MSC studies showed that not all patients benefit from MSC therapy, the principal efficacy of MSC is still under debate [2]. Hence, it is very likely that the success rate may also vary for therapies using MSC-EVs treatment.

We hypothesize that both the immune-modulatory capabilities of distinct MSC-EV preparations and the individual patients’ responsiveness towards MSC-EVs are critical factors for a successful therapeutic application of MSC-EV preparations. Thus, the objectives of our study were (i) to establish an inflammation associated in vitro assay characterizing MSC-EV functionality considering T cell differentiation via the expression of the homing molecule CCR7, the surface marker CD45RA and the proinflammatory IFNγ and TNFα cytokine response and (ii) to investigate the repercussion of individual recipient responsiveness on MSC-EV functionality. To address these issues, we used phorbol 12-myristate 13-acetate (PMA) and Ionomycin as reagents for the in vitro assay to mimic inflammation and immune cell response of T cell subsets.

## 2. Results

### 2.1. Induction of T cell Differentiation and Cytokine Production Upon PMA/Ionomycin Stimulation

**Figure 1:**
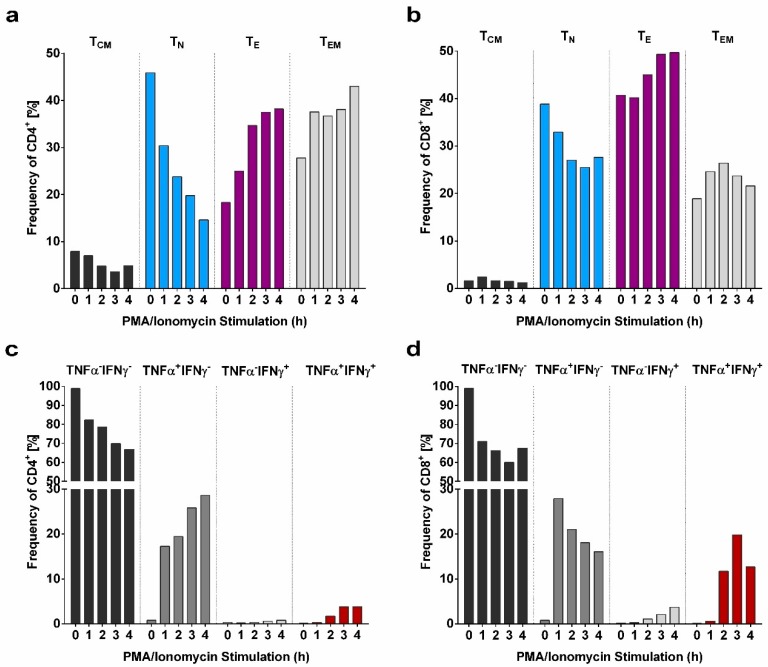
Influence of phorbol 12-myristate 13-acetate (PMA)/Ionomycin stimulation on T cell subsets and cytokine response over course of time. (**a**,**b**) Shift of the frequencies of CD4^+^ and CD8^+^ central memory (T_CM_), naïve (T_N_), effector (T_E_), and effector memory (T_EM_) T cell subsets upon PMA/Ionomycin stimulation for 1–4 h. (**c**,**d**) Modification of the TNFα and IFNγ response of CD4^+^ and CD8^+^ cells within four hours of PMA/Ionomycin stimulation.

To track the shift of T cell subsets and their corresponding TNFα and IFNγ response from a steady state towards a differentiated state in a time dependent manner, PMA/Ionomycin stimulation was performed for 0, 1, 2, 3, and 4 h (hours) for one donor sample (Figure 1). Importantly, stimulation did not affect cell viability as the frequency of living cells remained constant over time. T cells can be distinguished into naïve T cells (T_N_), central memory T cells (T_CM_), effector T cells (T_E_) and effector memory T cells (T_EM_). The general gating strategy employed to define the T cell subpopulations is depicted in S2. The phenotypical definition of T cell subsets via CCR7 and CD45RA expression revealed a decline in the frequencies of less differentiated T cell subsets T_N_ (CD45RA^+^CCR7^+^) and T_CM_ (CD45RA^−^CCR7^+^) and increasing frequencies of CD4^+^ and CD8^+^ T_E_ (CD45RA^+^CCR7^−^) reaching a plateau after 4 h of stimulation (Figure 1a,b). The frequencies of CD4^+^ and CD8^+^ T_EM_ cells (CD45RA^−^CCR7^−^) were increased at all time points of PMA/Ionomycin stimulation.

**Figure 2:**
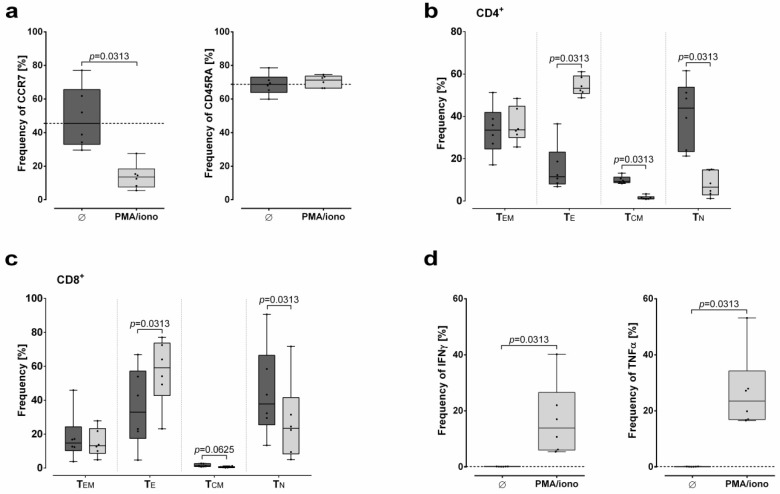
Influence of PMA/Ionomycin stimulation on T cell differentiation and cytokine response. Effect of PMA/Ionomycin stimulation for 4 h on (**a**) the surface expression of CCR7 and CD45RA, differentiation of (**b**) CD4^+^ and (**c**) CD8^+^ naïve (T_N_), central memory (T_CM_), effector (T_E_), and effector memory (T_EM_) T cell subsets, and (**d**) the cytokine response. Frequencies of cell populations (%) are presented as median with minimum and maximum. Black dotted line indicates the median frequency of a certain population obtained without PMA/Ionomycin stimulation. Statistical analysis was performed by Wilcoxon test.

The analysis strategy to determine the cytokine response of the T cell subsets is visualized in S3. In accordance with the phenotypical definition of T cell subsets over time, substantial frequencies of TNFα producing CD4^+^ and CD8^+^ cells were detectable as early as after 1 h stimulation (Figure 1c,d). Of note, within the CD8^+^ T cells, frequency of TNFα producing cells declined with time, while frequency of IFNγ producing cells increased. In contrast, frequency of TNFα producing CD4^+^ cells increased with time. As shown in Figure 2, PMA/Ionomycin in vitro stimulation of PBMC (*N* = 6 donors) for 4h clearly allows the evaluation of T cell responsiveness towards their competence of differentiation and cytokine production.

### 2.2. Modulation of CD45RA Expression on T cells by MSC-EVs upon PMA/Ionomycin Stimulation

**Figure 3:**
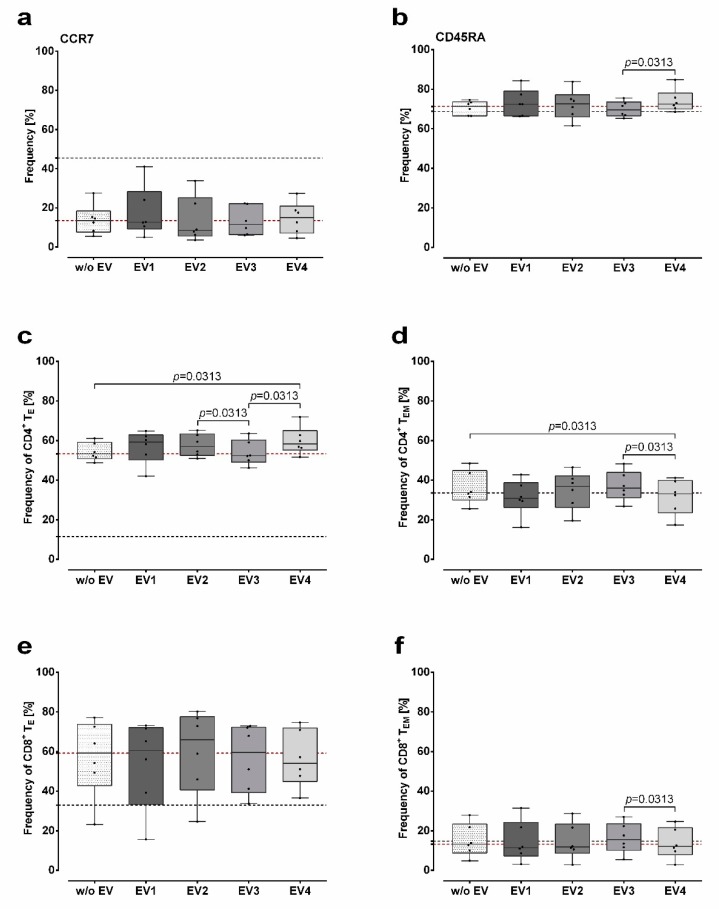
Modulation of CD45RA expression on T cells by MSC-EVs. (**a**,**b**) Effect of four different mesenchymal stem/stromal cells–extracellular vesicles (MSC-EV) preparations (EV1–EV4) on the frequency of CCR7 and CD45RA expressing T cells and (**c**–**f**) capacity to modulate CD4^+^ and CD8^+^ effector (T_E_) and effector memory (T_EM_) T cell subsets upon 4 h of PMA/Ionomycin stimulation. Frequencies of cell populations (%) are presented as median with minimum and maximum. Black dotted line indicates the median frequency of a certain population obtained without PMA/Ionomycin stimulation; red dotted line indicates the median frequency of a certain population obtained after PMA/Ionomycin stimulation in the absence of EV. Statistical analysis was performed by Wilcoxon test.

We have characterized the marker expression of the MSC-EV preparations by Western blot (S1). The presence of MSC-EVs does not lead to a further reduction of frequencies of T cells expressing CCR7 than the stimulation with PMA/Ionomycin alone (Figure 3a). No differences in frequencies of CCR7 expressing T cells were observed among the miscellaneous MSC-EV preparations. Contrary to impacts on the CCR7 expression, the presence of selected MSC-EV preparations during stimulation affected CD45RA expression on PBMCs: increased frequency of CD45RA expressing PBMCs were observed in the presence of MSC-EV4 compared to MSC-EV3 (*p* = 0.03, Figure 3b). Obviously, the presence of MSC-EV1 did not substantially affect the CD45RA expression.

Accordingly, further stratification of T cells into T_N_, T_CM_, T_E_, and T_EM_ cells revealed that the presence of MSC-EV4 during PMA/Ionomycin stimulation resulted in an increased frequency of CD4^+^ T_E_ compared to the stimulated control and to stimulated cells in presence of MSC-EV3 (*p* = 0.03; Figure 3c). Additionally, frequency of CD4^+^ T_E_ was increased in the presence of MSC-EV2 compared to the one of MSC-EV3 (*p* = 0.03). A reversed effect was observed for the frequencies of CD4^+^ T_EM_ (Figure 3d): presence of MSC-EV4 during stimulation reduced the frequency of CD4^+^ T_EM_ compared to the stimulation without MSC-EVs (*p* = 0.03) or in the presence of MSC-EV3 (*p* = 0.03). In CD8^+^ T cells frequency of CD8^+^ T_EM_ (*p* = 0.03; Figure 3f), but not of CD8^+^ T_E_ (Figure 3e) differed between cells stimulated in the presence of MSC-EV4 and MSC-EV3. The frequencies of T_N_ or T_CM_ in both, CD4^+^ and CD8^+^ T cells, were not significantly affected by MSC-EVs (Figure S4). Taken together, these results indicate that certain MSC-EVs have the capacity to modulate the expression of the differentiation marker CD45RA on CD4^+^ and CD8^+^ T cells, leading to a shift of T_EM_ and T_E_ frequencies upon PMA/Ionomycin stimulation.

### 2.3. Modulation of the Cytokine Response of T cells Subsets by MSC-EVs upon PMA/Ionomycin Stimulation

**Figure 4:**
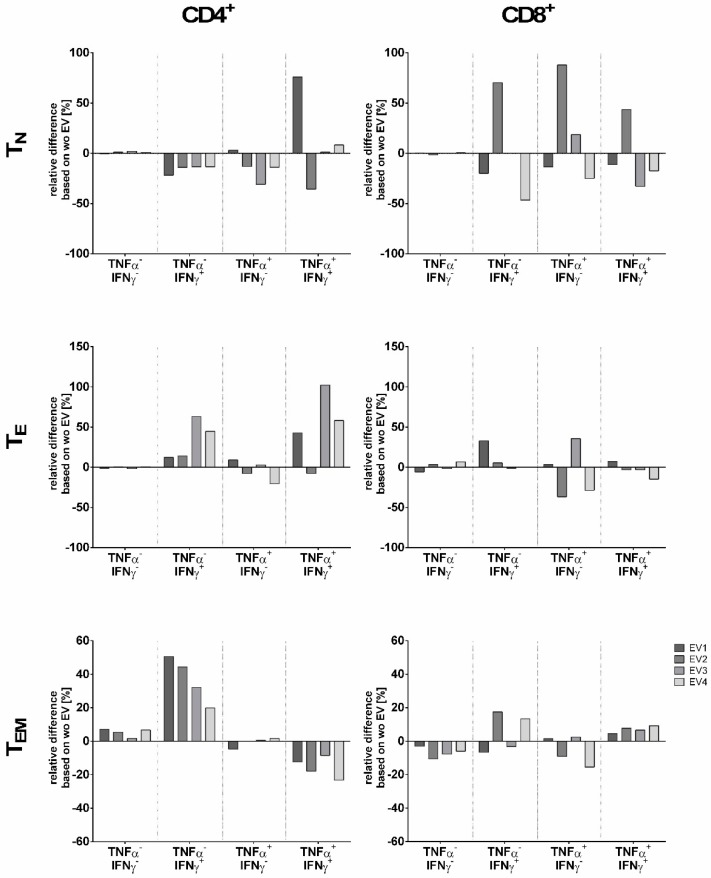
Modulation of the cytokine response of T cells subsets by MSC-EVs. Effect of different MSC-EV (EV1–EV4) on the proinflammatory cytokine distribution pattern of CD4^+^ and CD8^+^ naïve (T_N_), effector (T_E_) and effector memory (T_EM_) T cell subsets upon 4 h of PMA/Ionomycin stimulation. Given are the relative differences normalized to the stimulation in absence of MSC-EV.

Regarding the functional consequence of the presence of MSC-EVs during stimulation, the TNFα and IFNγ production profile in CD4^+^ and CD8^+^ T cells subsets was investigated (Figure 4). To allocate the cytokine response within the T_N_, T_CM_, T_E_, and T_EM_ subsets, distribution of TNFα^−^IFNγ^−^, TNFα^+^IFNγ^−^, TNFα^−^IFNγ^+^, and TNFα^+^IFNγ^+^ producing cells within a given T cell subset was analyzed. To this end, frequencies of cytokine producing cells obtained from stimulations in the presence of MSC-EVs were normalized to the ones obtained without MSC-EVs. For all EV preparation, CD4^+^ T_N_ showed reduced frequencies of cells producing exclusively IFNγ. At variance to this, increased frequencies of IFNγ producing CD4^+^ T cells belonging to the T_E_ and T_EM_ subsets were observed. However, these two subsets differed in their TNFα^+^IFNγ^+^ profile: for all MSC-EV preparation except MSC-EV2 increased frequencies of TNFα^+^IFNγ^+^ producing cells were found in the CD4^+^ T_E_ subset, whereas reduced frequencies of TNFα^+^IFNγ^+^ producing cells were observed in the CD4^+^ T_EM_ subset. Further, frequencies of cells lacking both, TNFα and IFNγ, were augmented in CD4^+^ T_EM_ subset.

Concerning CD8^+^ T cell subsets, it seemed that each EV preparation exhibited a unique modulation of the cytokine production in the CD8^+^ T_N_ subset: increased frequencies of cytokine producing cells (TNFα^−^IFNγ^+^, TNFα^+^IFNγ^−^, or TNFα^+^IFNγ^+^) were found for MSC-EV2 preparation, whereas reduced frequencies were observed for MSC-EV1 and MSC-EV4; the presence of MSC-EV3 did not cause a substantial variation of cytokine release in the CD8 T_N_ subset. For CD8^+^ T_E_ no distinct distribution pattern of frequencies were observed for TNFα^+^IFNγ^−^, TNFα^−^IFNγ^+^, or TNFα^+^IFNγ^+^ producing cells in context with a certain EV preparation. In comparison to CD4^+^ T_EM_ subset, CD8^+^ T_EM_ subset showed an opposed cytokine profile with increased frequencies of TNFα^+^IFNγ^+^ producing cells and reduced frequencies of cells lacking TNFα and IFNγ. Taken together, on one hand we observed homogenous effects of the four MSC-EV preparations considering that all MSC-EVs increased the frequency of TNFα^−^IFNγ^+^ producing cells in CD4^+^ T_E_ and T_EM_ subsets, and decreased the frequency of TNFα^−^IFNγ^+^ producing cells in CD4^+^ T_N_ and TNFα^+^IFNγ^+^ producing cells in CD4^+^ T_EM_. On the other hand, we observed opposing effects regarding TNFα^−^IFNγ^+^ and TNFα^+^IFNγ^−^ in CD8^+^ T_N_ and TNFα^+^IFNγ^−^ in CD8^+^ T_E_ subsets. These results illustrate the specific effect of various MSC-EVs on cytokine production of immune cells.

### 2.4. Influence of PBMC Donor Heterogeneity during MSC-EV Administration Upon PMA/Ionomycin Stimulation

**Figure 5:**
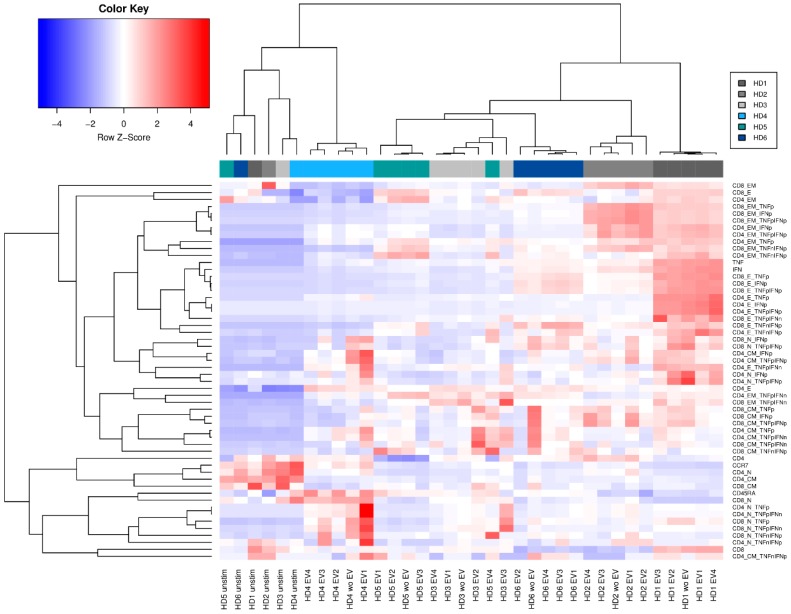
Hierarchical clustering regarding individual peripheral blood mononuclear cells (PBMC) responsiveness to MSC-EV. Clustering was performed with all factors included in the flow cytometric analysis. Compared was the effect of four different MSC-EV preparations (EV1–EV4) on PBMCs from six healthy donors (HD1–HD6).

To investigate whether PBMC of different donors respond in a similar or different manner to MSC-EVs, PBMCs derived from six healthy donors were PMA/Ionomycin stimulated, either in the presence or in absence of the four different MSC-EV preparations. Following flow cytometric analyses of the treated PBMCs in which we investigated the presence of the different T cell subsets, we performed hierarchical clustering of the frequencies of all T cell subpopulations (Figure 5). All unstimulated controls clustered together. Healthy donor 1 (HD1) showed the strongest response towards the PMA/Ionomycin stimulation compared to the unstimulated controls. With the exception of healthy donor 5 (HD5) all samples of each donor clustered together, independent of whether and which MSC-EV preparation were applied. Apparently, PBMCs react in donor specific manners towards PMA/Ionomycin. This effect seems to dominate the immune-modulatory properties of the different applied MSC-EV preparations. Consequently, donor specific reactions have to be considered upon investigating the immune-modulatory potential of different MSC-EV preparations. Consequently, for therapeutic approaches it is highly recommended to analyze the recipient’s responsiveness towards a certain MSC-EV preparation intended to be applied prior to MSC-EV treatment.

## 3. Discussion

Since the first application by the group of Le Blanc in 2004 [1], MSC have been used as treatment option in therapy-refractory GvHD. However, response rates in studies varied significantly and the efficacy of MSC in GvHD treatment is still controversially discussed [2]. The inconsistent results are difficult to evaluate since the manufacturing of MSC, the dosage of MSC infused, the number and timing of infusions and other characteristics vary largely among the studies. More importantly, the physiological proof for the immune-modulatory efficacy of MSC is still a matter of debate [3]. The central questions persist: do all MSC have a similar immune-modulatory effect and do all patients treated with MSC have a similar responsiveness to MSC treatment. In recent years, it has been suggested that the beneficial immune-modulatory effect of MSCs derive rather from secreted vesicles than from MSCs themselves [4,5]. Our group successfully applied MSC-derived EVs in a case of therapy-refractory GvHD [6]. This innovative treatment option is supported by mouse models: the group of Lim showed that MSC-derived EVs alleviates GvHD symptoms and increases survival which was associated with a significant increase in CD4^+^CD25^+^CD127^low/−^ regulatory T cells [7]. Another mouse model confirmed that MSC-derived EVs reduced GvHD damage in target organs and prolonged survival [8].

In light of the central questions of the efficacy of MSCs and responsiveness of patients to MSCs and in order to facilitate further applications of MSC-derived EVs in inflammatory processes, it was the objective of this study to establish a functional in vitro assay allowing testing for the immune-modulatory capacities and cytokine secretion potential of individual MSC-EV preparations and to investigate the influence of recipient responsiveness on MSC-EV functionality. The results of our study demonstrated that PMA/Ionomycin in vitro stimulation of PBMC enables the observation of T cell differentiation and cytokine production and hence can serve as an assay to monitor T cell responses. Second, we could show that various MSC-EV have the capacity to modulate differentially the expression of CD45RA on CD4^+^ and CD8^+^ T cells, which results in shifts of T_E_ and T_EM_ frequencies. Third, the cytokine response of T cells is additionally influenced by the various MSC-EV preparations. Fourth, and most importantly, by hierarchal clustering of all stimulation results we could clearly demonstrate that the response towards specific MSC-EV is highly dependent of the recipient’s responsiveness towards MSC-EV.

Our assay places emphasis on the monitoring of T cell differentiation and corresponding cytokine responses. Indeed, presence of an EV preparation derived from a distinct MSC during PMA/Ionomycin stimulation resulted in increased frequencies of CD45RA expressing T_E_ cells and decreased frequencies of T_EM_. Here, it is tempting to speculate that the presence of this distinct MSC-EV preparation facilitates the re-expression of CD45RA in memory T cells. Such CD45RA re-expressing cells are known to display senescence-related proliferative defects that are reversible [9]. Recently, in an animal model a reverse effect was observed for EVs derived from rat bone marrow MSCs (RBMSCs) overexpressing indoleamine 2,3-dioxygenase (IDO-RBMSCs). The presence of IDO-RBMSCs-derived EVs but not common RBMSCs-derived EVs led to decreased expression of CD45RA in a T cell and dendritic cell culture [10]. Hence, the originating MSC and its status contribute to the modulation of CD45RA expression via EVs.

To characterize the functionality of T cell subsets, our assay additionally captures the effect of independent MSC-EV preparations on TNFα and IFNγ cytokine responses, as these cytokines are important mediators in a variety of inflammation-associated disorders, in particular in the pathogenesis of GvHD [11,12,13]. Although the application of MSC-derived EVs in a patient with therapy-refractory GvHD revealed a reduced proinflammatory cytokine profile in vitro and in vivo [6], it is not established which T cell subsets are preferentially responsible for the immune-modulatory effect obtained. In particular, serum levels may not be a valid reflection of the cytokine responsiveness of a certain T cell subset. Our first results obtained by the established assay clearly illustrate the specific effect of various MSC-EVs on the cytokine production of different immune T cell subsets. Of note, the presence of MSC-EV preparations differentially affected the CD4^+^ and CD8^+^ T cell subset responsiveness. This kind of information and the knowledge of T cell subsets being involved in certain inflammatory disorders may allow forecasting the therapeutic success of a distinct MSC-EV preparation.

Lastly and most importantly, we could illustrate varying responsiveness of certain MSC-EV preparations on various recipients: hierarchical clustering revealed that the response towards specific MSC-EVs is highly dependent on the recipient’s responsiveness towards MSC-EVs. Hence, the specific influence of a certain MSC-EV preparation on T cell differentiation and the cytokine production as well as the responsiveness of a given patient who is intended for treatment with MSC-EV for severe GvHD or any other severe inflammatory-associated disorder have to be considered prior to application in order to optimize treatment results.

In this regard, our established functional in vitro assay yields important results regarding the immune-modulatory potency of MSC-EVs. Thus, we provide a tool delivering rapidly relevant information regarding the immune-modulatory capacities of a given MSC-EV preparation towards T cell subsets and their corresponding cytokine production. Due to the novelty of the MSC-EV field’s internationally accepted guidelines for their clinical grade production, quality assurance and clinical application are still being developed [2]. Functional potency markers and easily deployable methods of measurement would benefit EV research and help to meet regulatory requirements [14]. To this end, reliable and easily performable functional potency assays are required. In view of these aspects, our results warrant further investigations to implement this kind of assay as a promising platform for quality assurance of EV functionality. The prediction of non-responders would circumvent ineffective treatments and help patients to receive alternative therapies that may be more effective and finally yet importantly could save costs. Overall, in the future, this might enable clinicians to select the most appropriate MSC-EV preparation for an individual patient to treat therapy-refractory GvHD or any other severe inflammatory-associated disorder, e.g., stroke or acute myocardial infarction. In the near future, we plan to validate this in vitro potency assay in blood samples collected from patients after alloSCT with and without severe GvHD.

## 4. Materials and Methods

### 4.1. Isolation and Characterization of Extracellular Vesicles from Mesenchymal Stem Cells

MSCs were isolated from bone-marrow aspirates of four healthy individuals after informed consent as described before [6]. Approval of the Ethical Committee of the University Hospital Essen was obtained (12-5295-BO, 17.01.2013 and 25.06.2018). Cell culture supernatants (conditioned media; CM) were collected for EV purification by differential centrifugation and PEG precipitation as recently described [15]. For details, see supplementary information. MSC-EV fractions were studied (i) by nanoparticle tracking analysis on the ZetaView (Particle Metrix, Meerbusch, Germany) to define size and particle concentration (Table S1) [16], (ii) by protein assay (Thermo Scientific, Darmstadt, Germany) to define the protein concentration (Table S1) and (iii) by SDS PAGE and western blot to verify the expression of components associated with EVs including TSG101 (Sigma-Aldrich, St. Lois, MO, USA), Syntenin (clone EPR8102; Abcam, Cambridge, UK), CD81 (clone 5A6; Biolegend, San Diego, CA, USA), CD9 (VJ1, kindly provided by Francisco Sánchez-Madrid) and the absence of cytochrome C (clone 6H2.B4; Biolegend) to exclude cellular protein contamination as previously recommended [17,18] as minimal requirement for definition of EVs (Figure S1).

### 4.2. Stimulation of PBMC

Frozen PBMCs of healthy donors (HD) (for isolation and storage of PBMC see supplementary information) were carefully thawed and thereafter cultured in a 96-U-bottom plate in complete medium consisting of RPMI-1640 (Thermo Fisher Scientific, Darmstadt, Germany), 10% human AB serum (Transfusion Medicine, University Hospital Essen, Germany), 1% penicillin/streptomycin (Thermo Fisher Scientific, Darmstadt, Germany) and 5% CO_2_ in a density of 0.6 × 10^6^ cells/well in a total volume of 200 µL at 37 °C. Cells were stimulated with a cocktail of 32 nM PMA (Merck KGaA, Darmstadt, Germany) and 3.2 µM Ionomycin (Merck KGaA, Darmstadt, Germany) over a different length of time given in the text. To detect intracellular cytokines, the intracellular protein transport inhibitors BrefeldinA (10 µM; Thermo Fisher Scientific, Darmstadt, Germany) and Monensin (2 µM; Thermo Fisher Scientific, Darmstadt, Germany) were additionally included in culture medium during cell stimulation either in absence or in presence of different MSC-EV preparations. Stimulation was performed with a fix protein amount of 40 µg.

### 4.3. Flow Cytometric Analysis

Cell viability was analyzed by staining with LIVE/DEAD™ Violet Dead Cell Stain Kit according to manufacturer’s instructions (ThermoFisher Scientific, Darmstadt, Germany). Surface expression of T cells was analyzed by staining with fluorchromes-conjugated mononuclear antibodies targeting CD3 (BV510 clone OKT3), CD4 (ECD clone T4), CD8 (PerCP-Cy5.5 clone SK1), CD45RA (PE, clone HI100) and CCR7 (AF700 clone G043H7). All antibodies were provided by BioLegend (Koblenz, Germany) with the exception of CD4 (Beckman Coulter, Krefeld, Germany). Cells were fixed and permeabilized using the Foxp3/Transcription Factor Staining Buffer Set (ThermoFisher Scientific, Darmstadt, Germany) according to manufacturer’s instructions. Intracellular staining was accomplished by staining with fluorchromes-conjugated mononuclear antibodies targeting IFNγ (IFNγ BV421 clone 4S.B3) and TNFα (TNFα BV605 clone MAb11), both provided by BioLegend. Isotype matched antibodies served as negative controls (BD Bioscience, Heidelberg, Germany). Samples were subjected to multicolor flow cytometry using a CytoFlexS cytometer (Beckman Coulter, Krefeld, Germany). Data acquisition was accomplished with CytExpert Version 2.1 software (Beckman Coulter, Krefeld, Germany). Data analysis was performed by Kaluza Analysis 2.0. General gating is visualized in Figure S2, and an overview of the analysis strategy is given in Figure S3.

### 4.4. Statistical Analysis

Statistical analysis was performed by using GraphPad Prism V6.0 software (GraphPad Software, San Diego, CA, USA). Frequency of cell populations (%) is presented as median with minimum and maximum. Continuous variables were compared by student *t*-test and nonparametric Wilcoxon test after testing for Gaussian distribution. *p*-values < 0.05 were considered to be statistically significant. Hierarchal clustering was performed by R version 1.1.442 using the g-plots [19,20,21]. Reproducibility and overall variability was established by the method of bootstrap (n = 100).

## Supplementary Materials

Supplementary materials can be found at https://www.mdpi.com/1422-0067/20/7/1642/s1. Figure S1: Characterization of MSC-EV preparations by Western blot, Figure S2: General gating strategy to define T cell subpopulations in PBMC, Figure S3: Overview of the analysis strategy to determine the cytokine response of T cell subsets, Figure S4: Influence of MSC-EVs on T_N_ and T_CM_ subsets.
