# Supplementary material for: Individual Immune-Modulatory Capabilities of MSC-Derived Extracellular Vesicle (EV) Preparations and Recipient-Dependent Responsiveness
Source: Int J Mol Sci. 2019 Apr 2;20(7):1642. doi: 10.3390/ijms20071642 (PMC6479947; doi:10.3390/ijms20071642)
Supplement: Supplementary file 1 [file ijms-20-01642-s001.zip › 2019-03-07 Supplementary Figure Legends.docx]

**Supplementary Figure Legends**

**S1: Characterization of MSC-EV preparations by Western blot.** EV marker expression analysis for Cytochrome C, TSG101, Syntenin, CD9, and CD81 in four different MSC-EV preparations (MSC-EV 1-4). THP-1 cell lysate (C) served as a control.

**S2: General gating strategy to define T cell subpopulations in PBMC.**

**S3: Overview of the analysis strategy to determine the cytokine response of T cell subsets.**

**S4: Influence of MSC-EVs on T_N_ and T_CM_ subsets.** Effect of different MSC-EV on the frequency of CD4^+^ and CD8^+^ T_N_ and T_CM_ upon 4h of PMA/Ionomycin stimulation. Frequencies of cell populations [%] are presented as median with minimum and maximum. Black dotted line indicates the median frequency of a certain population obtained without PMA/Ionomycin stimulation; red dotted line indicates the median frequency of a certain population obtained after PMA/Ionomycin stimulation in the absence of EV.
