# Supplementary figures and images for: Individual Immune-Modulatory Capabilities of MSC-Derived Extracellular Vesicle (EV) Preparations and Recipient-Dependent Responsiveness
Source: Int J Mol Sci. 2019 Apr 2;20(7):1642. doi: 10.3390/ijms20071642 (PMC6479947; doi:10.3390/ijms20071642)

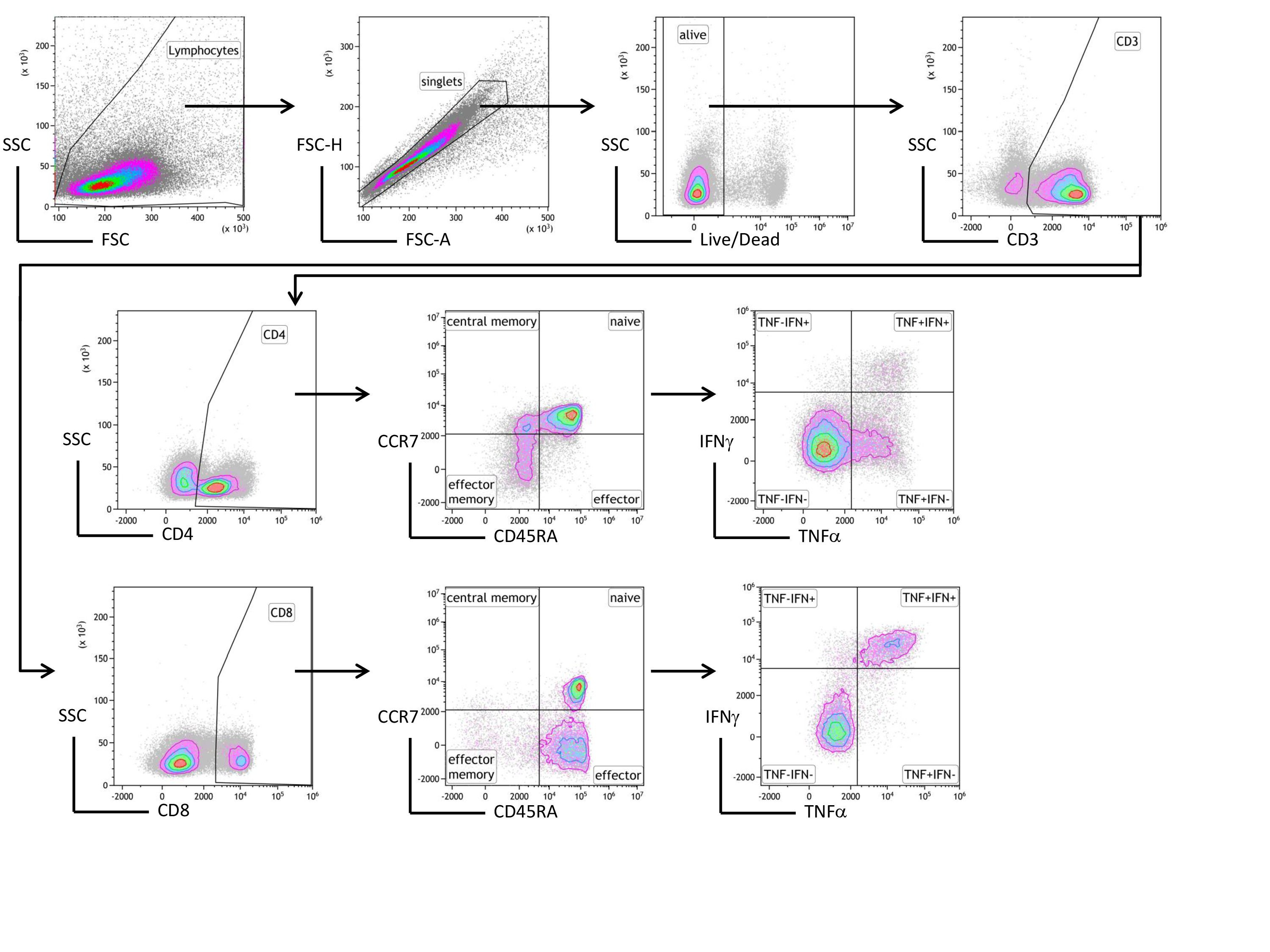

Supplement: Supplementary file 1 [file ijms-20-01642-s001.zip › 2019-03-07 S2 Gating Strategy.tiff]

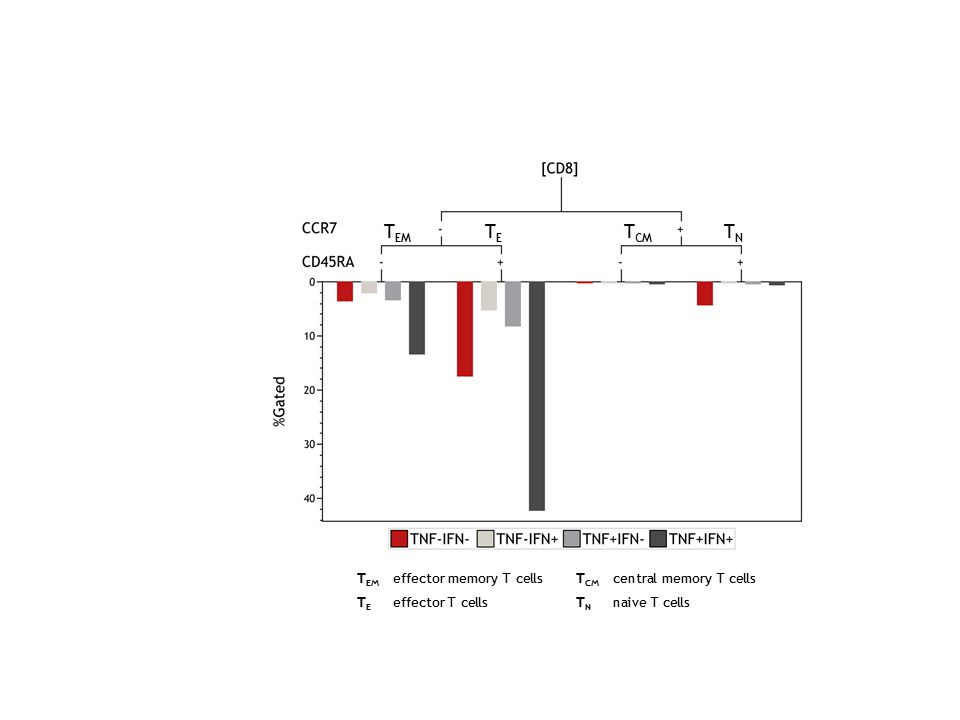

Supplement: Supplementary file 1 [file ijms-20-01642-s001.zip › 2019-03-07 S3 Analysis Strategy.jpg]

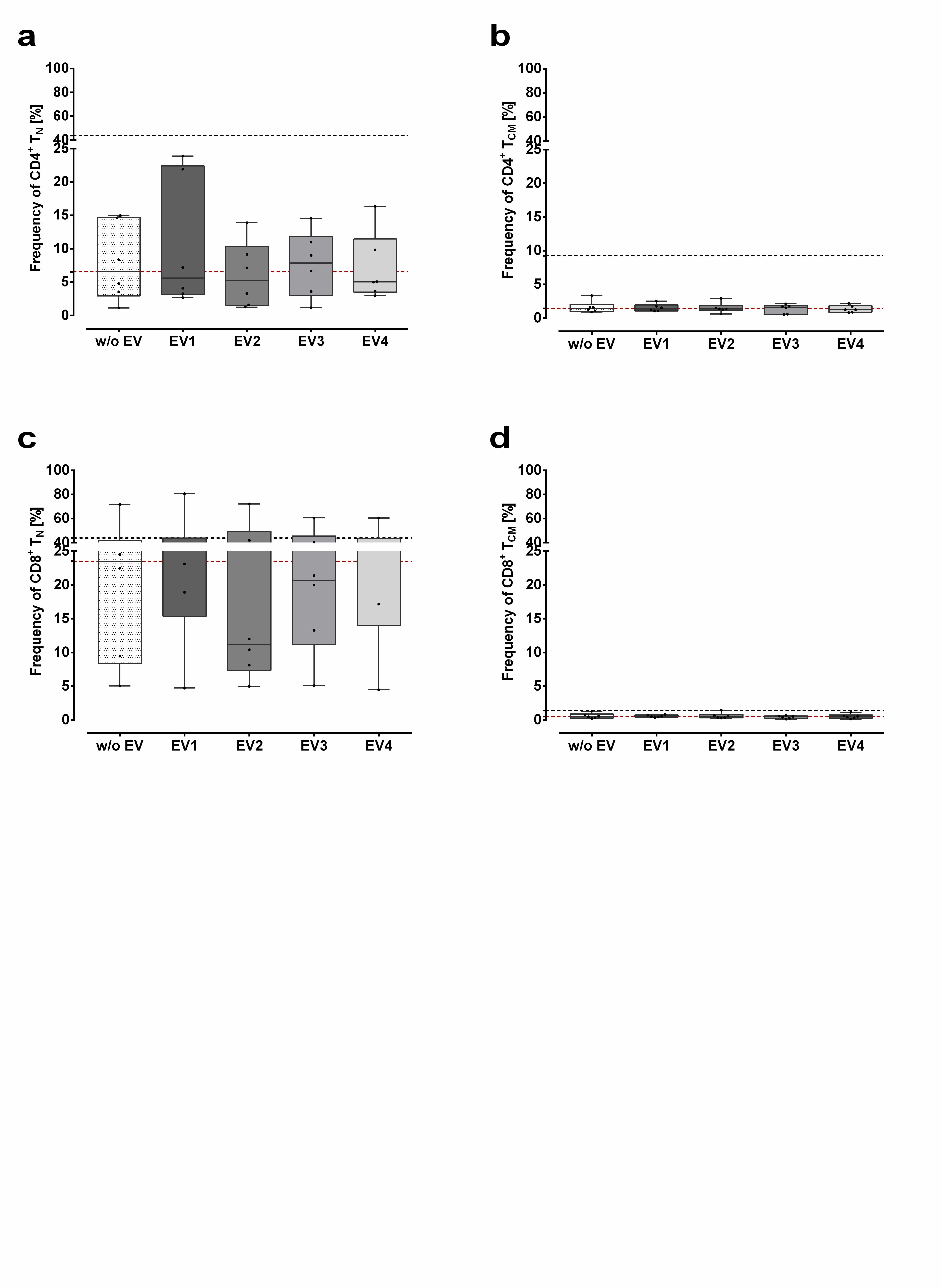

Supplement: Supplementary file 1 [file ijms-20-01642-s001.zip › 2019-03-07 S4 N und CM.jpg]

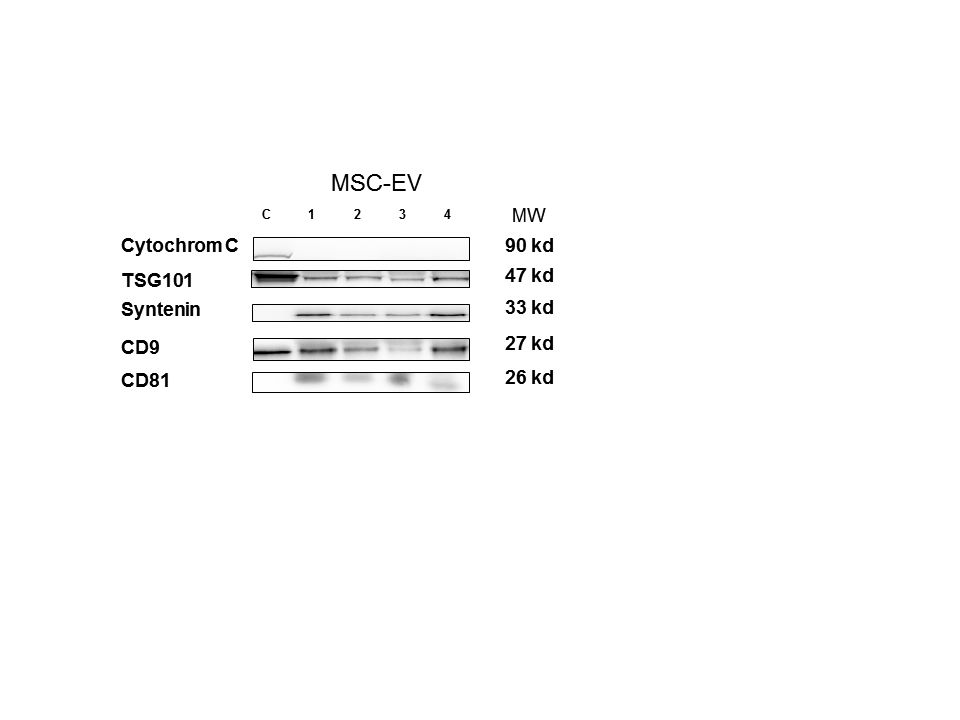

Supplement: Supplementary file 1 [file ijms-20-01642-s001.zip › 2019-03-07 S1 Western Blot EV marker.jpg]
